# Transportation of thermal and velocity slip factors on three-dimensional dual phase nanomaterials liquid flow towards an exponentially stretchable surface

**DOI:** 10.1038/s41598-022-21966-y

**Published:** 2022-11-03

**Authors:** Azad Hussain, Nevzat Akkurt, Aysha Rehman, Haifaa F. Alrihieli, Fahad M. Alharbi, Aishah Abdussattar, Sayed M. Eldin

**Affiliations:** 1 Department of Mathematics, University of Gujrat, Gujrat, 50700 Pakistan; 2 Department of Mechanical Engineering, Munzur University, 62000 Tunceli, Turkey; 3 Department of Mathematics, Faculty of Science, University of Tabuk, P.O. Box 741, Tabuk, 71491 Saudi Arabia; 4 Department of Mathematics, Al-Qunfudah University College, Umm Al-Qura University, Mecca, Saudi Arabia; 5 Center of Research, Faculty of Engineering, Future University in Egypt, New Cairo, 11835 Egypt

**Keywords:** Engineering, Mathematics and computing, Physics

## Abstract

The fundamental purpose of this research is to elaborate on slip boundary conditions and the flow of three-dimensional, stable, incompressible, rotating movements of nanoparticles lying across a stretchable sheet. The mathematical model for fluid flow is created using the assumptions stated above. The partial differentials are produced after utilizing boundary layer estimates. The partial differential governing equations are reduced into three coupled ordinary differential equations by using similarity transformations. After, applying transformations the system is solved numerically. Numerical results are approved with the help of the MATLAB bvp4c algorithm. The analysis shows that velocity and temperature are strongly dependent on essential parameters like stretching ratio, velocity slip, rotation, thermal slip parameter, and Prandtl number. Numerical values of distinct parameters on heat flux and skin friction factors are shown in a tabulated form. Partial velocity and thermal slip are applied to the temperature surface. The comparison among the nano-sized particles copper oxide and silver with water base nanofluid affecting velocity and temperature fields are used for analysis. Moreover, the Graphical depiction designates that the velocity and temperature spreading of the thermal slip parameter is increasing. It is observed that Ag-water is the best heat carrier as compared to CuO-water nanofluid.

## Introduction

The exploration of heat transfers plus boundary level movement through an extending surface is necessary for industrial as well as engineering technology. Types of alike flows include metallurgy, precipitation liquid films, wire drawing, hot rolling, produced filaments, etc. In all these cases, the final item feature is based happening the factor of friction and heat instability^1,2^. In these flows, the surface is often expected to be extended linearly. Nonetheless, in the real world, the stretching surface does not need to be linear as contended by Gupta et al.^3^. In most industrial and mechanical applications, heat transfer, which is assumed to be the most important process, has a constraint due to the essentially weak ordinary liquids of thermal conductivity. Therefore, significant attempts have been taken to resolve this constraint after Maxwell worked more than a hundred years ago on combining particles with a millimeter or micrometer scale in fluid^4–7^. As technology progress and power demand increase then the world needs to identify modern apparatus and devices that improve thermal dissipation. Heat flux has various industrial utilization of heat transfer that deal with temperatures that rise and fall equally. Due to their lower thermal conductivity, most fluids are not good heat conductors. To address this problem and improve these liquids' conductivity or other heat characteristics, a newly developed technique is used that involves the introduction of nanoparticles that are excellent heat conductors, such as titanium, iron, and aluminum through fluids^8^.

Choi was the one who initially coined the term "nanofluids"^9^. A nanofluid is a solution that includes a combination of nanoparticles. Nanoparticle characteristics exhibit a great deal of refinement at low nanoparticle concentrations. Nanoparticles are naturally formed in carbides, oxides, and carbon oxide tubes, which are employed in nanofluids. Base fluids are often ethylene glycol, water, plus thin oil. Understanding the behavior of nanofluids Hoghoughi et al.^10^ examined several models using a combination of nano-sized particles in a liquid. Wong and Long^11^ have explored the numerous tenders of nanofluids. grinding, domestic refrigerators, pharmaceutical processes, space technology, drug delivery, and nuclear reactor coolant by targeting especially copper nanoparticles that improve abnormal heart expansion and rotten arteries that have been established as a non-invasive technique to encounter heart diseases^12^. Numerous researchers have theoretically and experimentally explored the thermo-physical assets of nanofluid in the previous two decades. Several articles have demonstrated that the temperature rate transmission is higher than with typical base liquids^13–30^.

The main assumption of the Navier–Stokes concept is the no-slip border rule. Besides, this rule does not apply in some cases, such as in liquids, polymer solutions, emulsions, and foams. Whenever a fluid particle may occur near the extending border, partial slip velocity is applied. The situation of velocity slip occurs when the liquid to solid boundary flow is not applied evenly, which was examined in the light of specific conditions by wang^31^. After that, many researchers^32–40^ examined the problems, which were identified for velocity slip conditions at the boundary. The impression of velocity slip and temperature energy happening in the magneto- hydrodynamic mixture ${Cu-Al}_{2}{O}_{3}/$ water movement of nanofluid through a porous stretched area was investigated by Wahid et al.^41^. The phenomena of suspended atoms interacting with fluid across a flexible spinning disc are investigated by Turkyilmazoglu^42^. Also, Turkyilmazoglu explored the wall extending revolving fluxes in inertial and spinning contexts are considered in magnetohydrodynamics^43^.

The liquids that show boundary slip have an inclusive variety of uses, including a rubbing of implanted heart valves plus interior chambers. Mukhopadhyay studied thermal dissipation stratified MHD flows caused by an exponentially stretches sheet^44^. Thermophoretic particle deposition was used to explore a three-dimensional mixture of nano-fluid flow over a revolving expansion/contraction disc observed by Gowda et al.^45^. Jayadevamurthy et al.^46^ introduced attention to irregular mechanics of bioconvective mixed nanofluid flow across a rotating disc that moves uphill and downhill. Kotresh et al.^47^ explored the taxation of Arrhenius motivation energy in the overextended flow of nanofluid above a spinning disc. The influence of a realistic magnetic field happening the flow of liquid suspension among two revolving stretchy discs was studied by Radhika et al.^48^. The Cattaneo–Christov heat flux model was used by Gireesha et al.^49^ to study the MHD flow and molten heat transfer of grubby Casson fluid across a stretched sheet. Agrawal et al.^50^ studied the magneto Marangoni flow of γ − AL2O3 nanofluids above a stretching surface embedded in the porous medium through thermal radiation and heat source/sink effects. Khan et al. ^51^ introduced the stability analysis of dual solutions of nanomaterial flow comprising titanium alloy (Ti_6_, Al_4_ V) suspended in Williamson fluid through a thin moving needle with nonlinear thermal radiation. Iqbal et al.^52^ scrutinized the effect of induced magnetic field on thermal enhancement in gravity-driven Fe_3_O_4_ ferrofluid flow through the vertical non-isothermal surface. Chabani et al.^53^ demonstrated hybrid MHD nanofluid flow in a triangular enclosure. Over a stretched sheet, a rotating flow of MHD hybrid nanoparticles with heat radiation is investigated numerically by Shoaib et al.^54^. For innovative COVID-19 dynamics, a stochastic numerical study based on hybrid NAR-RBFs networks nonlinear SITR model was performed by Shoaib et al.^55^. Many researchers work on nanofluids in their studies^56–63^.

As a result, our goal is to make single-particle nanofluids with better characteristics. A revolving solution (Ag or CuO / water) causing slippage and thermal effects are analyzed in the present paper. The inspiration of velocity and thermal slip on rotating nanofluid and both types of nano-particles in three-dimensional flow is a unique component of this comparative research. Silver and copper oxide are exploited as nanoparticles, using water as the base fluid. The slippage velocity and thermal effects of this mixture of Ag-water and CuO-water nanoparticles have never been discussed in the literature. For the physical modeling of structure, the dual-phase model of nanofluid is used. The partial variance equations are concentrated to one that is easier to understand. The numerical results are achieved using Matlab and the bvp4c approach, a limited difference algorithm that executes the three-stage Lobatto method. The quality of velocity and temperature profiles is visually represented by various values of significant parameters.

## Problem formulation

**Figure 1:**
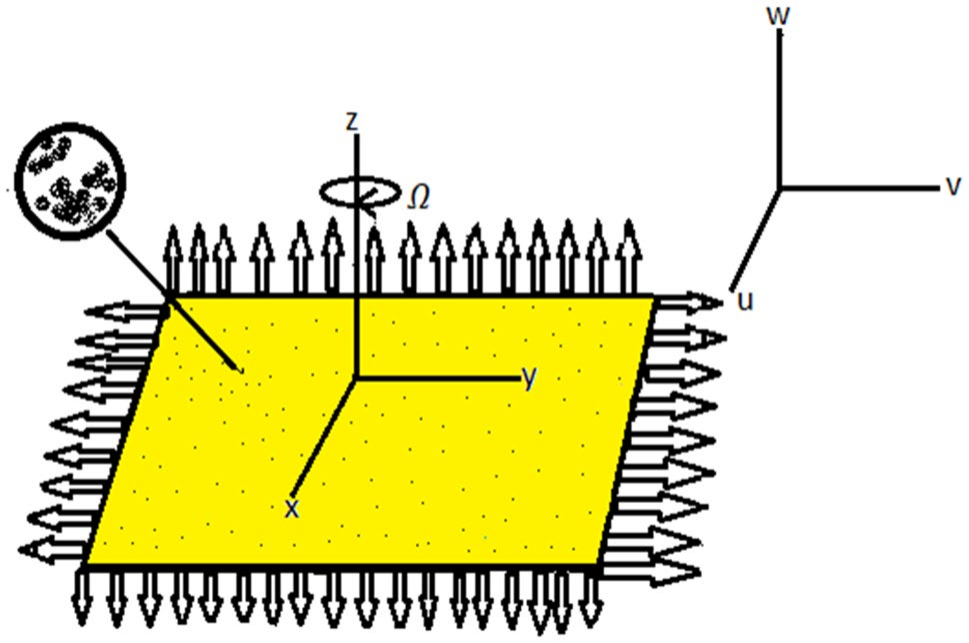
Geometry of the problem.

Let us Consider a steady, incompressible, three-dimensional, laminar rotating flow on a surface that is exponentially extending. Figure 1 describe the geometry of the problem. The flow is rotated around the z-axis at a constant angular velocity in the area $z\ge 0$. Exponentially surface is stretched with velocity ${u}_{w}$ and ${v}_{w}$. Copper oxide and silver are nano-sized elements with base liquid water (H_2_O) are considered for analysis. The governing three-dimensional equations^64^ become simpler when the boundary layer approximation is used and the pressure gradient and viscous dissipation are taken into account.

Continuity equation

1 $$\frac{\partial u}{\partial x}+\frac{\partial v}{\partial y}+\frac{\partial w}{\partial z}=0$$

Momentum equations

2 $$u\frac{\partial u}{\partial x}+v\frac{\partial u}{\partial y}+w\frac{\partial u}{\partial z}-2\Omega v={\nu }_{nf}\frac{{\partial }^{2}u}{\partial {z}^{2}},$$

3 $$u\frac{\partial v}{\partial x}+v\frac{\partial v}{\partial y}+w\frac{\partial v}{\partial z}+2\Omega u={\nu }_{nf}\frac{{\partial }^{2}v}{\partial {z}^{2}},$$

Energy equation

4 $$u\frac{\partial T}{\partial x}+v\frac{\partial T}{\partial y}+w\frac{\partial T}{\partial z}={\alpha }_{nf}\frac{{\partial }^{2}T}{\partial {z}^{2}},$$

where ${\nu }_{nf}$ is a kinematic viscosity, ${\alpha }_{nf}$ is the diffusivity of heat, and *T* stands for the temperature of the nanofluid.

5 $${{v}_{nf}=\frac{{\mu }_{nf}}{{\rho }_{nf}}}, \alpha_{nf}=\frac{{k}_{nf}}{{(\rho {C}_{p })}_{nf}},$$

The limitations are as follows:

6 $$\left.\begin{array}{c}u={u}_{w}+k{\nu }_{f}\frac{\partial u}{\partial z}, v={v}_{w}+k{\nu }_{f}\frac{\partial v}{\partial z},\\ u={u}_{w}+k{\nu }_{f}\frac{\partial u}{\partial z}, v={v}_{w}+k{\nu }_{f}\frac{\partial v}{\partial z},\\ w=0, T={T}_{w}+l\frac{\partial T}{\partial z}, at z=0,\end{array}\right\}$$

The dimensionless quantities are

7 $${u}_{w}={u}_{0 }{e}^{\frac{x+y}{L}}, { v}_{w}={v}_{0 }{e}^{\frac{x+y}{L}}, { T}_{w}={T}_{\infty }+{T}_{0} {e}^{\frac{A(x+y)}{2L}}.$$

Suitable similarity transformations are given below

8 $$\left.\begin{array}{c}u={u}_{0 }{e}^{\frac{x+y}{L}}{f}^{^{\prime}}\left(\eta \right), v={u}_{0 }{e}^{\frac{x+y}{L}}{g}^{^{\prime}}\left(\eta \right),\\ w=-{\left(\frac{v{u}_{0}}{2L}\right)}^\frac{1}{2}{e}^{\frac{x+y}{2L}}\left\{f+g+\eta {f}^{^{\prime}}+\eta {g}^{^{\prime}}\right\},\\ T={T}_{\infty }+{T}_{0} {e}^{\frac{A\left(x+y\right)}{2L}}\theta \left(\eta \right), \eta =z{\left(\frac{{u}_{0}}{2vL}\right)}^\frac{1}{2}{e}^{\frac{x+y}{2L}}.\end{array}\right\}$$

The continuity equation is identically fulfilled after implementing the similarity transformations to Eqs. (1–5) whereas the momentum and energy equations become

9 $$\frac{1}{{\left( {1 - \phi } \right)^{2.5} \left( {1 - \phi + \phi \frac{{\rho_{s} }}{{\rho_{f} }}} \right)}}f^{\prime\prime\prime} + f^{\prime\prime}\left( {f + g} \right) - 2f^{\prime}\left( {f^{\prime} + g^{\prime}} \right) + 4\gamma g^{\prime} = 0,$$

10 $$\frac{1}{{\left( {1 - \phi } \right)^{2.5} \left( {1 - \phi + \phi \frac{{\rho_{s} }}{{\rho_{f} }}} \right)}}g^{\prime\prime\prime} + g^{\prime\prime}\left( {f + g} \right) - 2g^{\prime}\left( {f^{\prime} + g^{\prime}} \right) - 4\gamma f^{\prime} = 0,$$

11 $$\frac{1}{Pr}\frac{{\left( {\frac{{k_{nf} }}{{k_{f} }}} \right)}}{{\left( {1 - \phi + \phi \frac{{\left( {\rho C_{p} } \right)_{s} }}{{\left( {\rho C_{p} } \right)_{f} }}} \right)}}\theta^{\prime\prime} + \theta^{\prime}\left( {f + g} \right) - \theta A\left( {f^{\prime} + g^{\prime}} \right) = 0,$$

here

12 $$\gamma =\frac{{\Omega }_{0}L}{{u}_{0}}, Pr=\frac{{\left(\mu {C}_{p}\right)}_{f}}{{k}_{f}}.$$

The transformed boundary conditions are

13 $$\left. {\begin{array}{*{20}c} {f\left( 0 \right) = 0, g\left( 0 \right) = 0, f^{\prime}\left( 0 \right) = 1 + \alpha f\prime \prime \left( 0 \right),} \\ {g^{\prime}\left( 0 \right) = \lambda + \alpha g^{\prime\prime}\left( 0 \right), \theta \left( 0 \right) = 1 + \beta \theta^{\prime}\left( 0 \right), at \eta = 0,} \\ { f^{\prime} \to 0, g^{\prime} \to 0, \theta \to 0, as \eta \to \infty ,} \\ \end{array} } \right\}$$

where, $\alpha$ stands for velocity slip, $\lambda$ denotes by stretching ratio, $\beta$ represents the thermal slip parameter.

14 $$\alpha =k{\left(\frac{v{u}_{0}}{2L}\right)}^\frac{1}{2}{e}^{\frac{x+y}{2L}}, \lambda =\frac{{v}_{0}}{{u}_{0}}, \beta =l{\left(\frac{{u}_{0}}{2vL}\right)}^\frac{1}{2}{e}^{\frac{x+y}{2L}}.$$

The Nusselt number and skin friction factors are also described.

15 $${C}_{fx}=\frac{{\tau }_{wx}}{\frac{1}{2}{\rho }_{f}{{u}_{w}}^{2}}, {C}_{fy}=\frac{{\tau }_{wy}}{\frac{1}{2}{\rho }_{f}{{u}_{w}}^{2}}, {Nu}_{x}=\frac{x{q}_{w}}{{k}_{f }({T}_{w}-{T}_{\infty })}.$$

The wall shear stresses $({\tau }_{wx}, {\tau }_{wy})$ and heat flux ${(q}_{w})$ are expressed as

16 $$\left.\begin{array}{c}{\tau }_{wx}={\mu }_{nf}{(\frac{\partial u}{\partial z})}_{z=0},\\ {\tau }_{wy}={\mu }_{nf}{(\frac{\partial v}{\partial z})}_{z=0},\\ {q}_{w}=-{k}_{nf}({\frac{\partial T}{\partial z})}_{z=0}.\end{array}\right\}$$

Now using Eqs. (7–8) and (16) in (15), we get

17 $$\left.\begin{array}{c}\frac{1}{\sqrt{2}}{C}_{fx}{(R{e}_{x})}^\frac{1}{2}=\frac{1}{{(1-\phi )}^{2.5}}{f}^{^{\prime\prime} }\left(0\right),\\ \frac{1}{\sqrt{2}}{C}_{fy}{(R{e}_{x})}^\frac{1}{2}=\frac{1}{{(1-\phi )}^{2.5}}g^{\prime\prime} \left(0\right),\\ \sqrt{2}\frac{L}{x}{Nu}_{x}{(R{e}_{x})}^{-\frac{1}{2}}=-\left(\frac{{k}_{nf}}{{k}_{f}}\right){\theta }^{\mathrm{^{\prime}}}\left(0\right),\end{array}\right\}$$

where Re is the Reynolds number in the area.

## Numerical scheme

A numerical result of the problem is obtained by using the MATLAB bvp4c algorithm. The governing PDEs with high non-linearity are converted into ODEs as a result of appropriate transformations. Equations (9), (10), and (11) are combined to form a first-order linear equation system. To fulfill the asymptotic boundary criteria, appropriate initial guesses are established (13). The following are the new variables

18 $$\left.\begin{array}{c}f={y}_{1}, {f}^{^{\prime}}={y}_{2}, {f}^{^{\prime\prime} }={y}_{3}, g={y}_{4},\\ {g}^{^{\prime}}={y}_{5}, {g}^{^{\prime\prime} }={y}_{6}, \theta ={y}_{7}, {\theta }^{^{\prime}}={y}_{8}.\end{array}\right\}$$

Therefore, the corresponding Eqs. (9–11) becomes

19 $${y}_{3}^{^{\prime}}={\left(1-\phi \right)}^{2.5}\left(1-\phi +\phi \frac{{\rho }_{s}}{{\rho }_{f}}\right)\left(2{{(y}_{2})}^{2}+2{y}_{2}{y}_{5}-{y}_{3}{y}_{1}-{y}_{3}{y}_{4}-4\gamma {y}_{5}\right),$$

20 $${y}_{6}^{^{\prime}}={\left(1-\phi \right)}^{2.5}\left(1-\phi +\phi \frac{{\rho }_{s}}{{\rho }_{f}}\right)\left(2{y}_{5}{y}_{2}+2{{(y}_{5})}^{2}-{y}_{6}{y}_{1}-{y}_{6}{y}_{4}+4\gamma {y}_{2}\right),$$

21 $${y}_{8}^{^{\prime}}=(\frac{{k}_{f}}{{k}_{nf}})(1-\phi +\phi \frac{{\left(\rho {C}_{p}\right)}_{s}}{{\left(\rho {C}_{p}\right)}_{f}})\left(\mathrm{Pr}\right)\left(\mathrm{A}{y}_{7}({y}_{1}+{y}_{4})-{y}_{8}({y}_{2}+{y}_{5}\right),$$

with boundary conditions

22 $$\left.\begin{array}{c}{y}_{1}\left(a\right)=0, {y}_{2}\left(a\right)=1+\alpha {y}_{3}\left(a\right), {y}_{3}\left(a\right)={a }_{0},\\ {y}_{4}{\left(a\right)=0, y}_{5}\left(a\right)=\lambda +\alpha {y}_{6}\left(a\right), {y}_{6}\left(a\right)={b}_{0},\\ {y}_{7}\left(a\right)=1+\beta {y}_{8}\left(a\right),, {y}_{8}\left(a\right)={c}_{0},\\ {y}_{2}\left(b\right)=0, {y}_{5}\left(b\right)=0, {y}_{7}\left(b\right)=0.\end{array}\right\}$$

The results show how dimensionless variables like stretching ratio, rotation parameter, slippage, temperature slip parameter, and Prandtl number influence the number of skin factors and the ratio of heat flux for the nanoparticles of CuO as well as Ag-water. The following graphs and tables depict the answers to the current problem.

## Graphical outcomes and discussions

**Figure 2:**
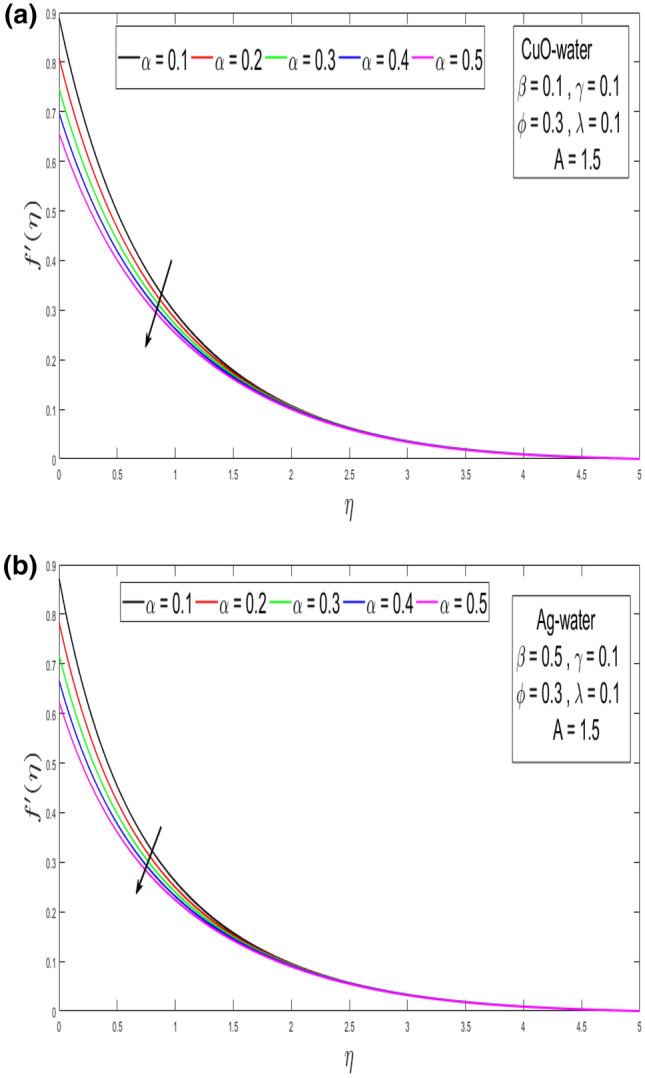
(**a**) Influence of velocity slip α parameter above $f^{\prime}\left( \eta \right)$ for CuO nanoparticle. (**b**) Influence of velocity slip α parameter over $f^{\prime}\left( \eta \right)$ for Ag nanoparticle.

**Figure 3:**
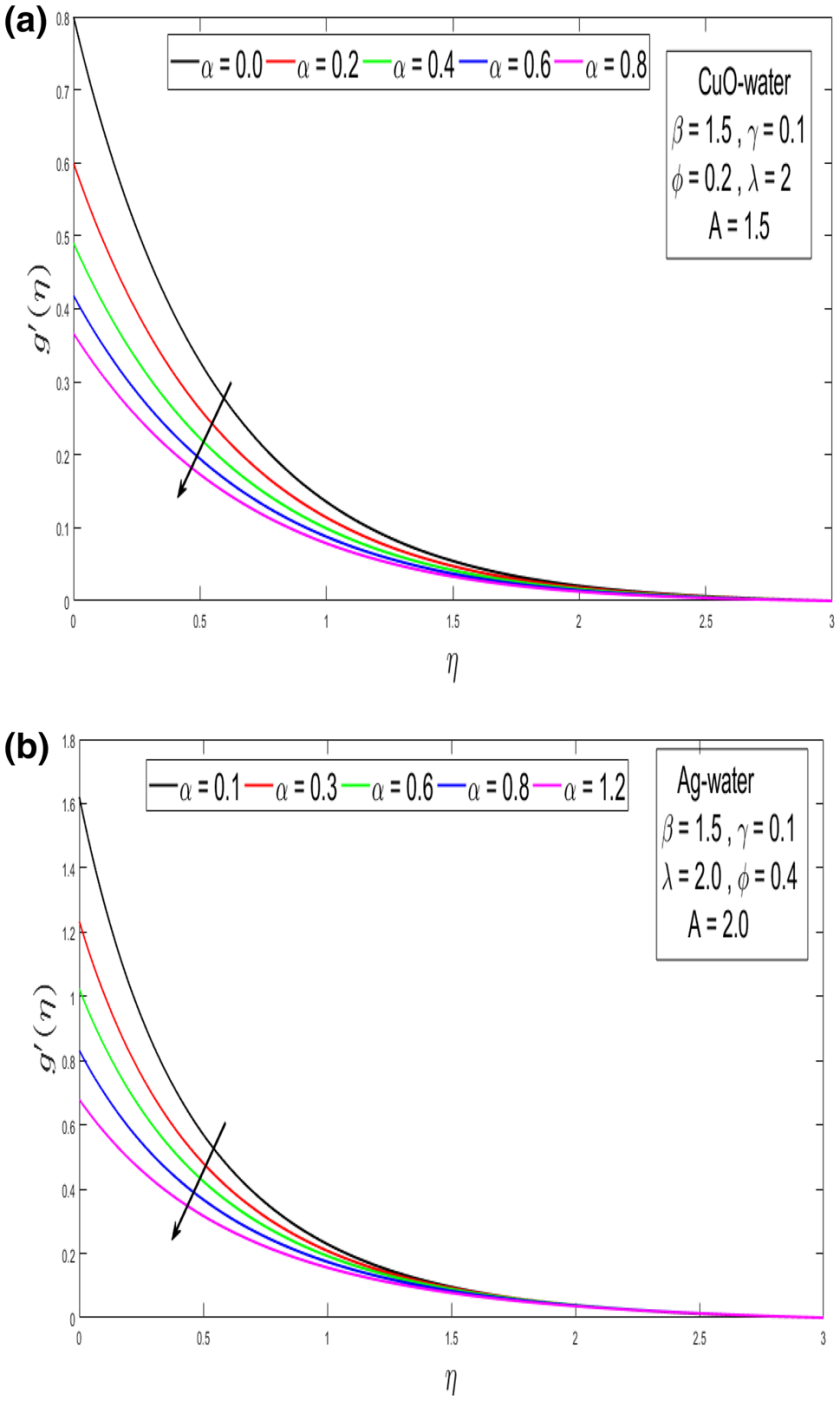
(**a**) Outcome of velocity slip α parameter over the velocity distribution $g^{\prime}\left( \eta \right)$ for CuO nanoparticle. (**b**) Outcome of velocity slip α parameter above the velocity distribution $g^{\prime}\left( \eta \right)$ for Ag nanoparticle.

**Figure 4:**
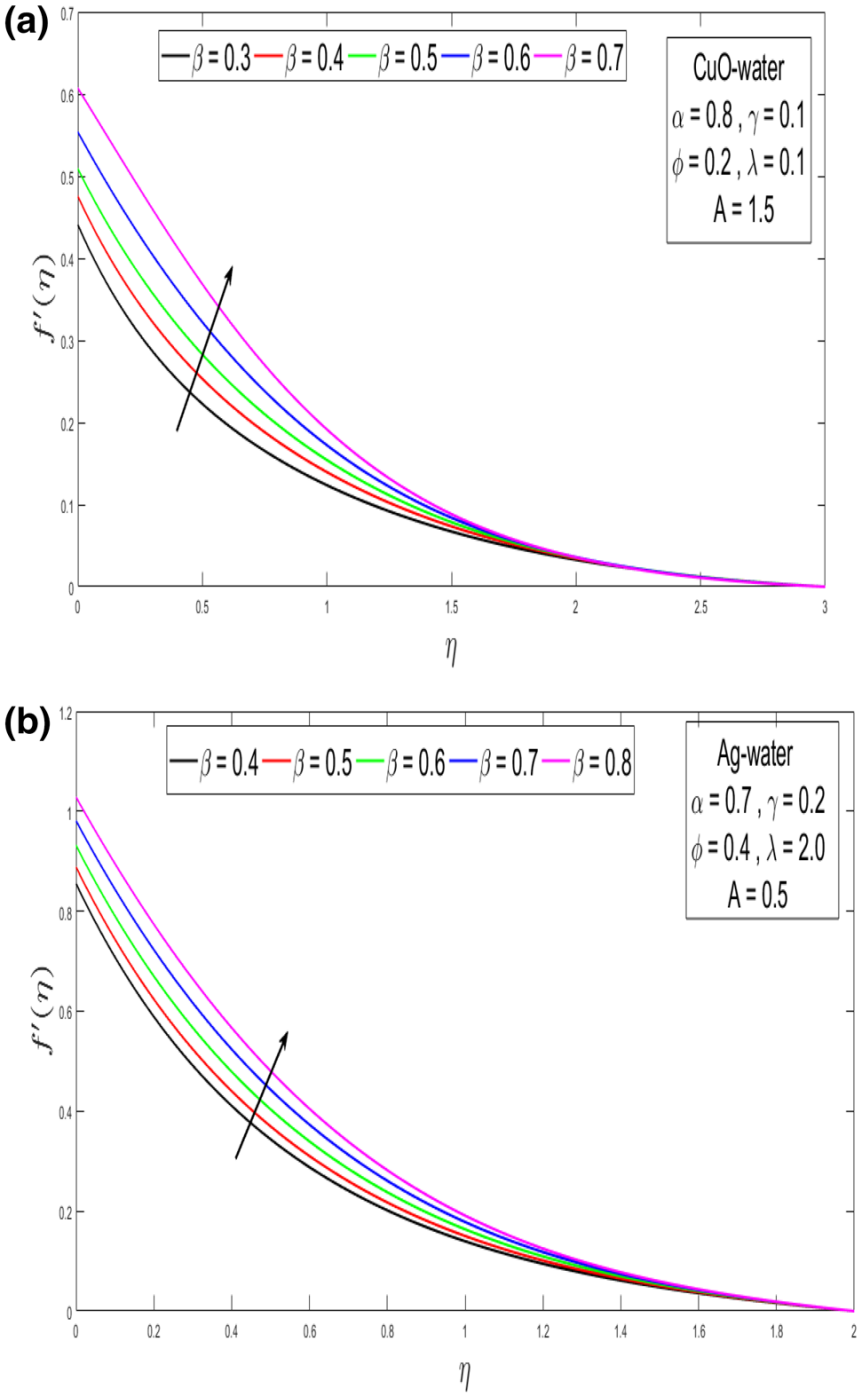
(**a**) Result of β over the velocity distribution $f^{\prime}\left( \eta \right)$ for CuO nanoparticle. (**b**) Result of β over the velocity distribution $f^{\prime}\left( \eta \right)$ for Ag nanoparticle.

**Figure 5:**
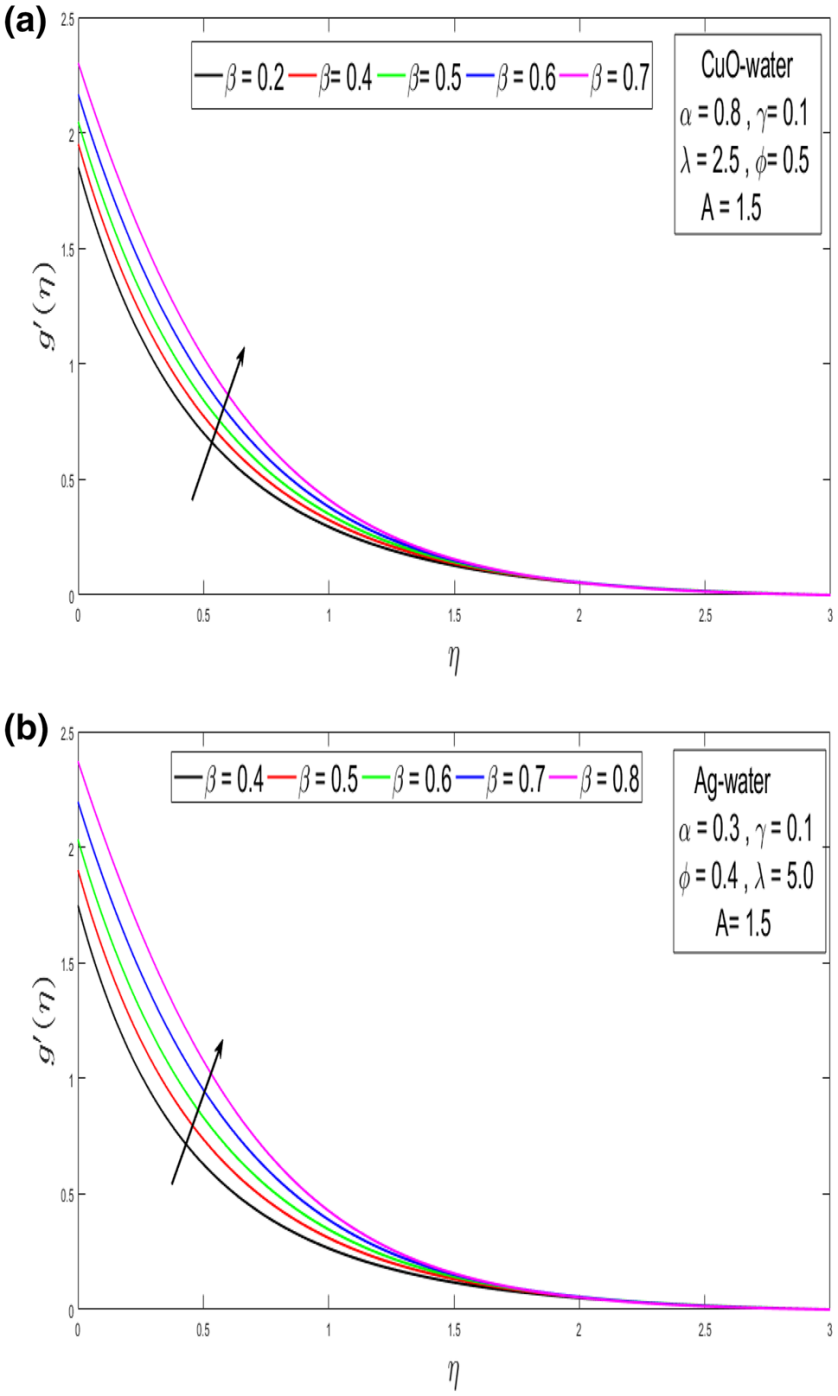
(**a**) Influence of thermal slip β parameter for CuO nanoparticle over the velocity distribution $g^{\prime}\left( \eta \right).$ (**b**) Influence of thermal slip β parameter for Ag nanoparticle above the velocity distribution $g^{\prime}\left( \eta \right)$.

**Figure 6:**
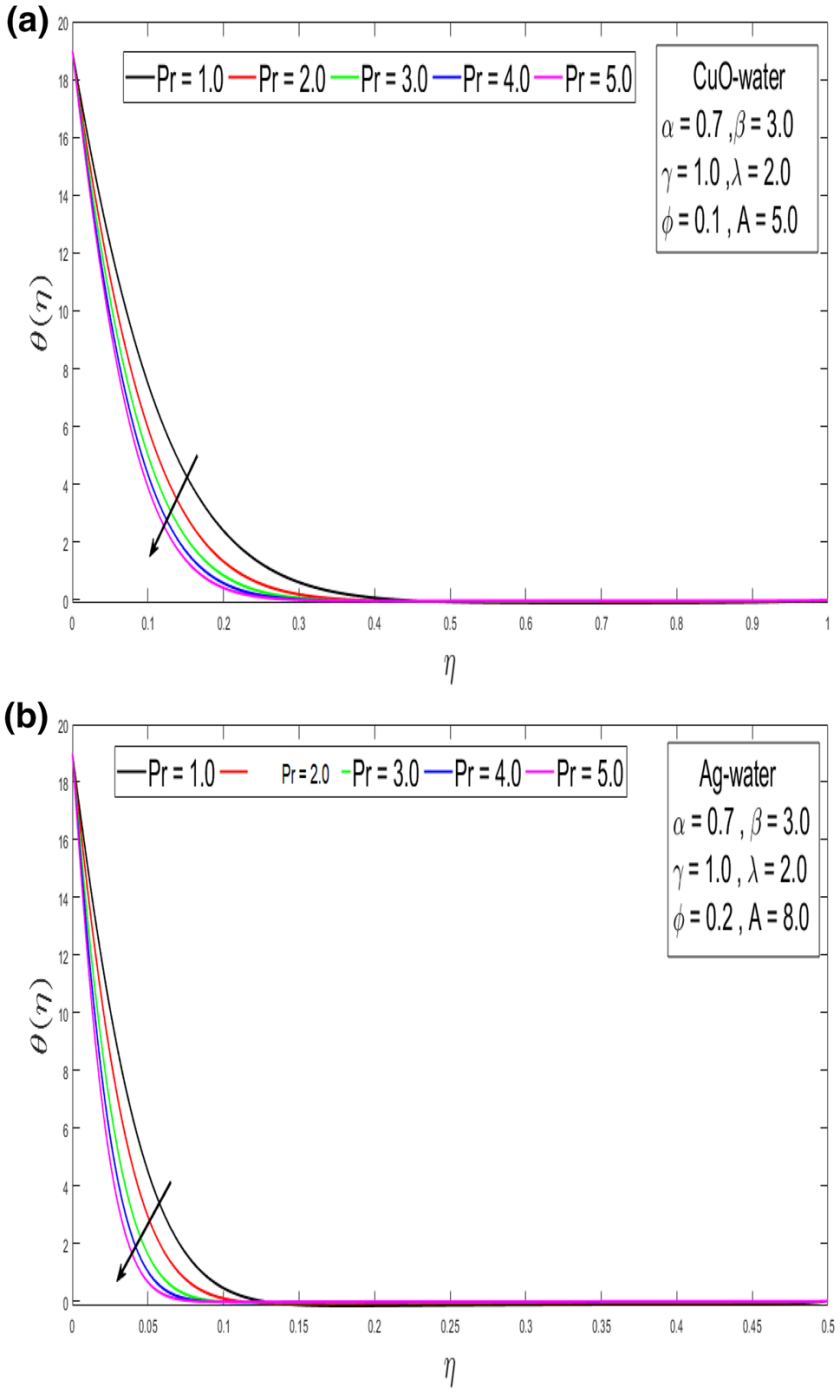
(**a**) Influence on Prandtl number (Pr) of θ(η)for CuO-water nanofluid. (**b**) Influence on Prandtl number (Pr) of θ(η) for Ag-water nanofluid.

**Figure 7:**
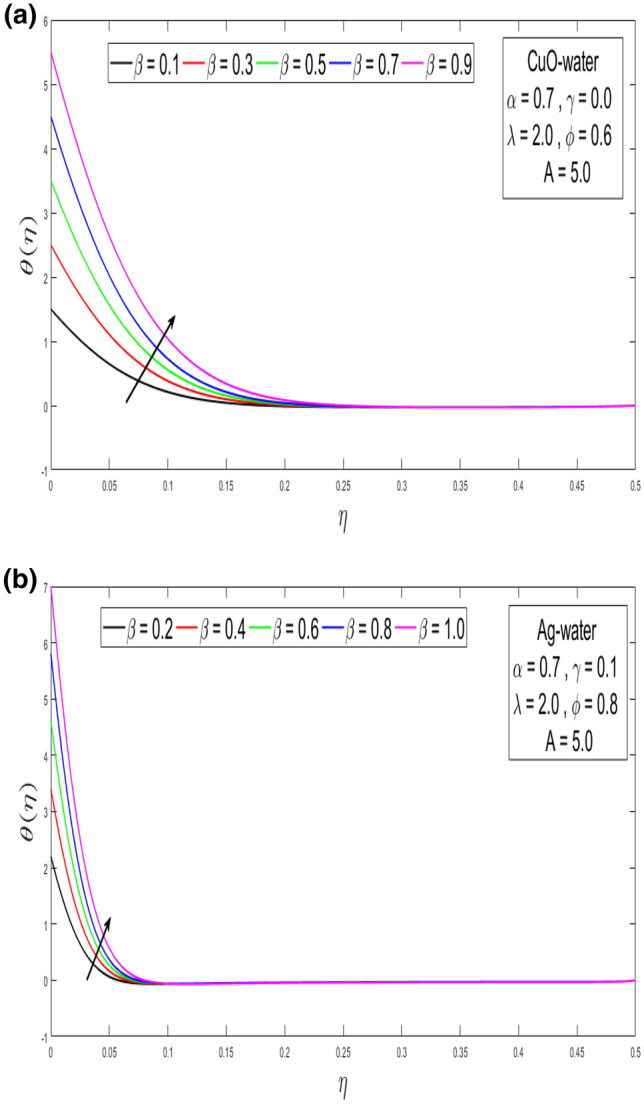
(**a**) Influence of temperature distribution θ(η) over thermal slip β parameter for CuO-water nanofluid. (**b**) Influence of temperature distribution θ(η) over thermal slip β parameter for Ag-water nanofluid.

**Figure 8:**
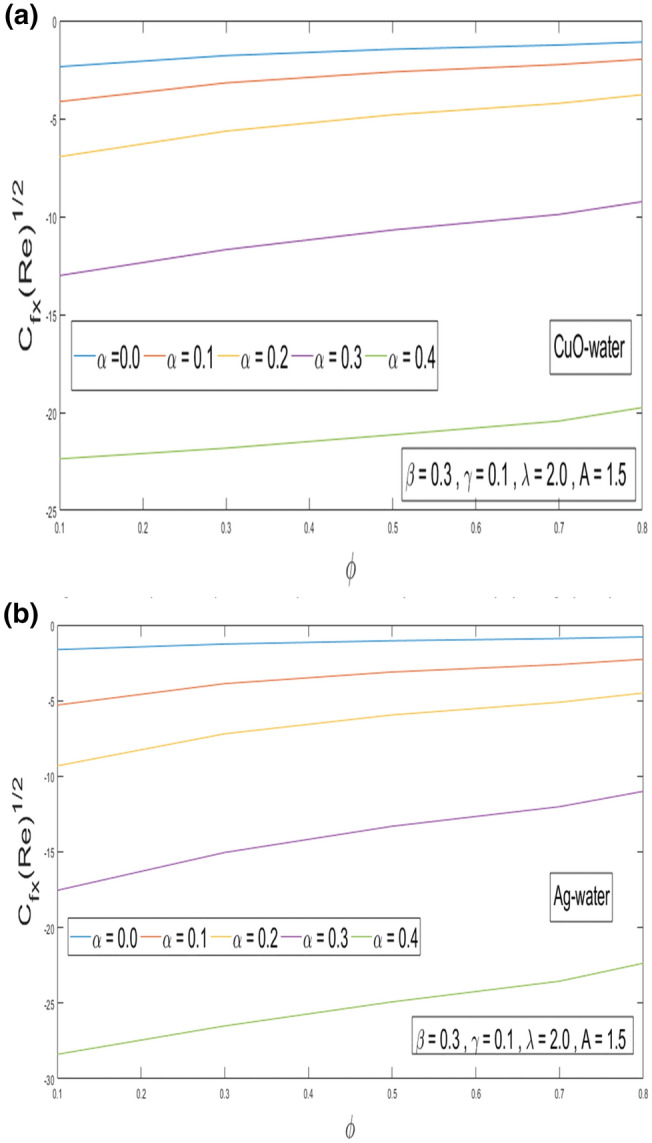
(**a**) The outcome of α and φ along the x-axis on skin friction for CuO nanoparticle. (**b**) The outcome of α and φ along the x-axis on skin friction for Ag nanoparticle.

**Figure 9:**
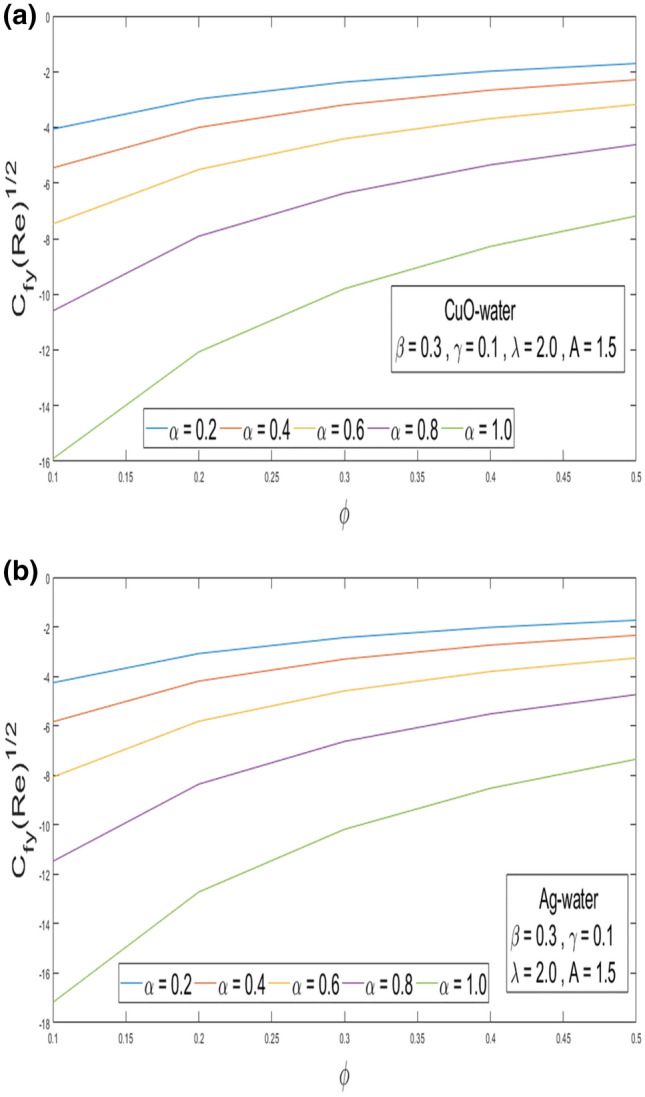
(**a**) Result of α and φ over ${C}_{fy}$ for CuO nanoparticle. (**b**) Result of α and φ above ${C}_{fy}$ for Ag nanoparticle.

**Figure 10:**
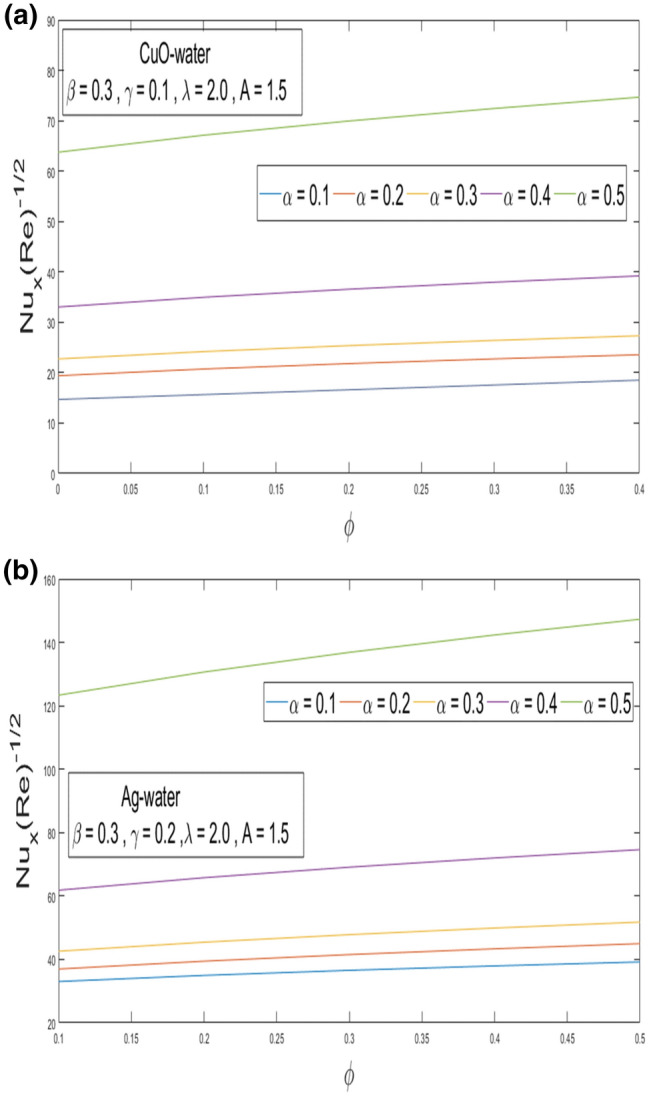
(**a**) Outcome of α and φ over Nusselt number for CuO-water of nanofluid. (**b**) Outcome of α and φ over Nusselt number for Ag-water of nanofluid.

This section looks at the effects of velocity in both directions $\left( {f^{\prime}\left( \eta \right), g^{\prime}\left( \eta \right)} \right)$ and thermal performance $\theta \left( \eta \right)$ for a few relevant parameters alike as velocity slip α, Prandtl number $Pr,$ thermal slip β for the CuO as well as Ag-water nanofluid. We obtain graphical findings in the existence of nanoparticles on velocity and temperature profiles that give the real influence of these parameters. Figure 2a,b are organized to reveal the influence of α on the velocity filed $\left( {f^{\prime}\left( \eta \right)} \right)$ for CuO and Ag-water solution. Figure 2a demonstrates the impression of the slip α parameter on the $f^{\prime}\left( \eta \right)$. While increasing the α, outcomes in a significant decline in the horizontal velocity distribution for CuO-water nanofluid. Also, Fig. 2b demonstrates that by growing of α parameter so the velocity curve is decreasing for Ag-water nanofluid. Figure 3a,b elaborate on the inspiration of the velocity slip α parameter along the vertical velocity field $\left( {g^{\prime}\left( \eta \right)} \right)$ for both types of nanofluids. Figure 3a represents that increases the values of α so the velocity profile shows downward for CuO-water nanofluid. From Fig. 3b, when an increasing velocity slip parameter α, the vertical velocity curve for Ag-water nanofluid is declined. This is because the speed of the molten nearby the surface in the existence of the slip does not remain as similar to the stretching surface velocity. Therefore, by increasing the α the slip velocity intensifies. It consequently reduces the velocity of the liquid since, below the slip form, the straining of the extending sheet can mostly be passed through the liquid. It is observed that the slip α parameter markedly influences the solutions. Similarly, the thickness of the boundary level declines as α is growing upward. In Figs. 4a,b and 5a,b expression that the influence of the thermal slip β parameter over the velocity distribution $\left( {f^{\prime}\left( \eta \right), g^{\prime}\left( \eta \right)} \right)$ are depicted for both types of nanoparticles with water-based. Figure 4a,b show that within growing the values of thermal parameter β, the velocity filed $\left( {f^{\prime}\left( \eta \right)} \right)$ is increased for both types of nanoparticles. In Fig. 5a,b denote that growing the values of β, then the velocity curve growing upward for Ag-water nanofluid. From Figs. 6a,b and 7a,b establish the impact of (Pr) and thermal slip parameter β over temperature element $\theta \left( \eta \right)$ for CuO-water and Ag-water. It can be shown that, from Fig. 6a,b, when increases Pr, then the temperature distribution is decline for both types of nanofluids. Figure 7a,b deliberate on the impression of thermal slip parameter β above the temperature $\theta \left( \eta \right)$ profile. This implies that through growing the rate of β on the temperature curve $\theta \left( \eta \right)$ increases for the CuO-water and Ag-water of nanofluids. Physically, additional heat is transferred from the surface toward the liquid with rising slip parameter β, which results in an increasing temperature profile. The distribution of temperature converges at 0.1 in the existence of the Ag-water and the temperature curve for CuO-water converges at 0.3. So, we observe that Ag-water is the best nanofluid as compared to CuO-water nanofluid. Physically, the CuO-water or Ag-water nanofluid has a comparatively upper thermal conductivity in production with the base fluid and nanoparticle. Figures 8a,b and 9a,b show the behavior of skin friction coefficient beside the x-axis $C_{fx} { }$ i.e. $\frac{1}{{\left( {1 - \phi } \right)^{2.5} }}f^{\prime\prime}\left( 0 \right),{ }$ and y-axis $C_{fy}$ i.e. $\frac{1}{{\left( {1 - \phi } \right)^{2.5} }}g^{\prime\prime}\left( 0 \right),$ with volume fraction φ for various morals of velocity slip α parameter by the boundary. It is analyzed from Fig. 8a,b display that the skin friction about x-direction is reduced with the rise in the velocity slip α and volume fraction φ. Also, denoted from Fig. 9a,b that within increased α parameter, the magnitude of friction amount $C_{fy}$ is declines for CuO-water as well as Ag-water. Figure 10a,b elaborates on the variation of velocity slip α with the volume fraction φ on the local heat flux. Here, it is recognized that the local thermal dissipation increases when an increase the value of volume fraction φ, velocity slip α parameter for CuO as well as Ag-water respectively. From these Figs. 8a,b, 9a,b and 10a,b, we observe that when velocity slip parameter α increases about x–y-direction, the length of skin friction decreases. But if we check the opposite, it has an opposing impact on the rate of thermal dissipation i.e. it allows the rate of heat dissipation to increase. Table 1, explicates the thermo-physical features of nanomaterials (CuO-water and Ag-water). Table 2, shows that the basic physical features of nanoparticles (CuO and Ag) and base liquid (H_2_O) are examined in the paper. Table 3, numerical values for coefficients of skin friction ($C_{fx}$,${ }C_{fy}$) and local thermal dissipation (Nu_x_ )for variations of velocity slip (α) parameter, rotation (γ) parameter, thermal slip (β) parameter, stretching ratio (λ) parameter, temperature exponent (A), volume fraction φ plus Prandtl number (Pr = 6.2) for CuO-water nanofluid. Table 3, the variation of distinct parameters α, γ, β, A, λ, and Pr = 6.2 on the quantity of skin friction $\left( {C_{fx} , C_{fy} } \right)$ and Nusselt number (Nu_x_ ) for the Ag-water of nanofluid. From Table 3, we notice that with growing the α, γ, and λ the magnitude of skin friction coefficient and heat flux increases at the surface. Also, in Table 4, when increasing the values of φ along with the variation of α, γ, β, A, and λ the magnitude of the coefficient of skin friction $\left( {C_{fx} , C_{fy} } \right)$ decreases but values of Nusselt number (Nu_x_) increase. However, when $\varphi$ increases, the value of heat dissipation increases since the thermal conductivity of nanomaterials is greater than the base liquid and these nanoparticles contain less specific heat than the base fluid. Table 5 describes the results of relevant factors on the magnitude of the velocity about the x-axis at the surface. The comparison in Table 5 demonstrates a high degree of concordance with previously reported data.

**Table 1:** Thermophysical properties of Nanofluids.

Properties	Nanofluids
Viscosity (μ)	${\mu }_{nf}=\frac{{\mu }_{f}}{{\left(1-\phi \right)}^{2.5}}$
Density (ρ)	${\rho }_{nf=}{\rho }_{f}\left(1-\phi \right)+\phi {\rho }_{s}$
Heat capacity $(\rho {C}_{p })$	${(\rho {C}_{p })}_{nf}={(\rho {C}_{p })}_{f}\left(1-\phi \right)+\phi {(\rho {C}_{p })}_{s}$
Thermal conductivity (K)	$\frac{{k}_{nf}}{{k}_{f}}=\frac{\left({k}_{s}+2{k}_{f}\right)-2\phi ({k}_{f}-{k}_{s})}{\left({k}_{s}+2{k}_{f}\right)+\phi ({k}_{f}-{k}_{s})}$

**Table 2:** Thermo-Physical Qualities of Nanoparticles (CuO, Ag) and Base Liquid H_2_O.

Properties	Density (ρ)	Thermal conductivity (K)	Specific heat (C_p_)
CuO	6320	76.50	513.80
Ag	10,500	429	235
H_2_O	977.1	0.6130	4179.0

**Table 3:** Numerical values of Distinct Parameters on Skin Friction $\left({C}_{fx}, {C}_{fy}\right)$ and Nusselt Number $\left({Nu}_{x}\right)$ for CuO-Water of Nanofluid for $Pr=6.2$.

$\alpha$	$\gamma$	$\lambda$	$\phi$	$\beta$	$A$	${C}_{fx}$	${C}_{fy}$	${Nu}_{x}$
0.0	0.3	0.2	0.1	0.2	0.5	−2.32639	−6.72241	17.53396
0.1						−1.76543	−5.03673	18.57086
0.2						−1.44410	−4.07737	19.39993
0.3						−1.23161	−3.44644	20.10707
0.4						−1.07902	−2.99556	20.73244
0.3	0.0	0.2	0.3	0.2	0.4	−1.57814	−3.15628	19.17597
	0.3					−1.24539	−3.43128	20.10599
	0.6					−1.01607	−3.73726	20.29480
	0.9					−0.88101	−4.02672	20.65244
	1.2					−0.80739	−4.27808	21.08388
0.4	0.0	0.2	0.0	0.3	0.5	−1.05330	−0.53299	25.19064
		0.3				−1.05586	−0.63359	24.02902
		0.4				−1.05740	−0.74166	23.09243
		0.5				−1.05959	−0.85592	22.31203
		0.6				−1.06183	−0.97546	21.64605
0.2	0.3	0.3	0.1	0.1	0.3	−1.45999	−4.06134	19.40120
			0.2			−1.96354	−5.45631	22.72750
			0.3			−2.61653	−7.47017	33.67590
			0.4			−3.51060	−10.5946	63.74770
			0.5			−4.80669	−15.9049	79.98456

**Table 4:** Variation of Distinct Parameters on Skin Friction $\left({C}_{fx}, {C}_{fy}\right)$ and Nusselt Number $\left({Nu}_{x}\right)$ for Ag-Water of Nanofluid for $Pr=6.2$.

$\alpha$	$\gamma$	$\lambda$	$\phi$	$\beta$	$A$	$\frac{1}{\sqrt{2}}{C}_{fx}{(R{e}_{x})}^\frac{1}{2}$	$\frac{1}{\sqrt{2}}{C}_{fy}{(R{e}_{x})}^\frac{1}{2}$	$\sqrt{2}\frac{L}{x}{Nu}_{x}{(R{e}_{x})}^{-\frac{1}{2}}$
0.0	0.3	0.2	0.1	0.2	0.5	−2.71438	−7.33084	33.80310
0.1						−1.99950	−5.35047	36.96885
0.2						−1.60832	−4.27170	39.44513
0.3						−1.35616	−3.57893	41.52940
0.4						−1.17813	−3.09144	43.35487
0.3	0.0	0.2	0.3	0.2	0.4	−1.66342	−3.32684	41.70715
	0.3					−1.36745	−3.56693	41.52735
	0.6					−1.15295	−3.83350	41.75213
	0.9					−1.01547	−4.09144	42.24726
	1.2					−0.93260	−4.32155	42.87038
0.4	0.0	0.2	0.0	0.3	0.5	−1.10540	−0.51490	52.97200
		0.3				−1.11931	−0.62117	50.46163
		0.4				−1.11881	−0.73525	48.38002
		0.5				−1.12396	−0.85574	46.60897
		0.6				−1.12944	−0.98166	45.07307
0.2	0.3	0.3	0.1	0.1	0.3	−1.04168	−4.25892	33.02740
			0.2			−2.27191	−5.83804	39.44610
			0.3			−3.10597	−8.06861	45.43090
			0.4			−4.25896	−11.4774	65.77500
			0.5			−5.94399	−17.1790	98.89052

**Table 5:** Comparison of ${-}f^{\prime\prime}\left( 0 \right)$ for different values of stretching ratio $\lambda$ when $\gamma$=0.0 = $\phi$.

$\lambda$	Wang (1984)	Ariel (2007)	Butt and Ali (2015)	Hayat (2019)	Present results
0.2	1.04180	1.03458	1.03949	1.04040	1.04150
0.3	1.06270	1.05247	1.05795	1.05871	1.05982
0.4	1.08360	1.07052	1.07578	1.07643	1.07732
0.5	1.10450	1.08866	1.09309	1.09364	1.09542

## Final remarks

This article analyzes the steady, three-dimensional boundary layer rotating nanofluid flow with partial and thermal slippage past an exponentially extending surface. Physical characteristics' effects on two side velocities, temperature field, and friction variables $\left({C}_{fx}, {C}_{fy}\right)$ and $({{\varvec{N}}{\varvec{u}}}_{{\varvec{x}}})$ are addressed comprehensively. The following are the major conclusions:

- Lateral directions $\left( {f^{\prime}\left( \eta \right), g^{\prime}\left( \eta \right)} \right)$ velocities are declines for growing the values of velocity slip α parameter.
- By raising the β parameter then the velocity profile along with two directions $\left( {f^{\prime}\left( \eta \right), g^{\prime}\left( \eta \right)} \right)$ velocities increases respectively.
- Silver (Ag) nanoparticle is better heat carriers as compared to Copper oxide (CuO) nanoparticle.
- The temperature curve and thermal boundary layer width rise while growing the thermal slip β parameter for both types of nanofluids.
- Temperature distribution θ(η) is decreasing while increasing Pr.
- By increasing the value of α, skin friction factors $({C}_{fx}, {C}_{fy})$ diminishes but heat flux rises at the surface.

## Data Availability

The datasets used or analysed during the current study available from the corresponding author on reasonable request.

## List of symbols

*K*: Velocity slip factor
$$\gamma$$: Rotation parameter
*u*_0_, *v*_0_: Rates of stretching
*C*_*fx*_, *C*_*fy*_: Skin friction along the x-direction and y-direction
*l*: Thermal slip factor
*Pr*: Prandtl number
*Nu*_*x*_: Nusselt number
*u*,*v*,*w*: Components of velocity in *x, y*, and *z*-direction
${T, T}_{w},{T}_{\infty }$: The temperature of the fluid, ambient temperature, wall temperature

## Greek Symbols

$\varphi$: Nano-particles volume fraction
$$\theta$$: Dimensionless temperature
$\Omega$: Constant angular velocity
$\eta$: Dimensionless space variable
$\upsilon_{f} \;,\,\;\upsilon_{nf}$: Kinematic viscosities of the base fluid and nanofluid respectively
$\mu_{f} \;,\,\;\mu_{nf}$: Dynamic viscosities of the base fluid and nanofluid respectively
$\rho_{s} \;,\,\;\rho_{f} \;,\,\;\rho_{nf\;\;\;\;\;\;\;\;}$: The density of solid nanoparticles, base fluid, and nanofluid respectively
$\alpha_{nf}$: Thermal diffusivity of nanofluid
$k_{s} \;,\,\;k_{f} \;,\,\;k_{nf}$: Thermal conductivities of solid nanoparticles, base fluid, and nano-fluid respectively
$(Cp)_{nf} \;,\,\;(Cp)_{f}$: Volumetric heat capacity of nanofluid and base fluid respectively
